# Fetal Therapy Model of Myelomeningocele with Three-Dimensional Skin Using Amniotic Fluid Cell-Derived Induced Pluripotent Stem Cells

**DOI:** 10.1016/j.stemcr.2017.05.013

**Published:** 2017-06-09

**Authors:** Kazuhiro Kajiwara, Tomohiro Tanemoto, Seiji Wada, Jurii Karibe, Norimasa Ihara, Yu Ikemoto, Tomoyuki Kawasaki, Yoshie Oishi, Osamu Samura, Kohji Okamura, Shuji Takada, Hidenori Akutsu, Haruhiko Sago, Aikou Okamoto, Akihiro Umezawa

**Affiliations:** 1 Department of Reproductive Biology, Center for Regenerative Medicine, National Research Institute for Child Health and Development, 2-10-1 Okura, Setagaya, Tokyo 157-8535, Japan; 2 Maternal-Fetal, Neonatal and Reproductive Medicine, National Research Institute for Child Health and Development, Tokyo 157-8535, Japan; 3 Systems BioMedicine, National Research Institute for Child Health and Development, Tokyo 157-8535, Japan; 4 Department of Obstetrics and Gynecology, The Jikei University School of Medicine, Tokyo 105-8471, Japan; 5 Department of Medical Science, Chiba University Graduate School of Medicine, Chiba 260-0856, Japan

**Keywords:** induced pluripotent stem cells, myelomeningocele, fetal therapy, keratinocytes, rock inhibitor, epidermal growth factor, amniotic fluid, polyhydramnion

## Abstract

Myelomeningocele (MMC) is a congenital disease without genetic abnormalities. Neurological symptoms are irreversibly impaired after birth, and no effective treatment has been reported to date. Only surgical repairs have been reported so far. In this study, we performed antenatal treatment of MMC with an artificial skin using induced pluripotent stem cells (iPSCs) generated from a patient with Down syndrome (AF-T21-iPSCs) and twin-twin transfusion syndrome (AF-TTTS-iPSCs) to a rat model. We manufactured three-dimensional skin with epidermis generated from keratinocytes derived from AF-T21-iPSCs and AF-TTTS-iPSCs and dermis of human fibroblasts and collagen type I. For generation of epidermis, we developed a protocol using Y-27632 and epidermal growth factor. The artificial skin was successfully covered over MMC defect sites during pregnancy, implying a possible antenatal surgical treatment with iPSC technology.

## Highlights

• Generation of iPS cells from patients with polyhydramnion
• Development of a novel differentiation protocol for iPSCs into keratinocytes
• Novel therapy model for myelomeningocele with artificial skin

## Teaser

In this article, Umezawa et al. obtained preclinical proof of concept for fetal therapy to patients with myelomeningocele (MMC) using autologous induced pluripotent stem cells from patients with Down syndrome and twin-twin transfusion syndrome. Our fetal cell treatment is minimally invasive and therefore has the potential to become a novel treatment for MMC.

## Introduction

Myelomeningocele (MMC) is the most common neural tube defect characterized by a skin defect in addition to defects of the midline vertebra and dura mater. Under these conditions, the spinal cord is exposed to the external environment. Although MMC is compatible with survival, this condition ranks as one of the most severe birth defects, with the manifestation of sequelae that affect both the central and peripheral nervous systems, leading to lifelong paralysis and dysfunction of the bladder and bowel for which no cures exist. The first human randomized controlled clinical trial entitled the “Management of Myelomeningocele Study (MOMS)” has found that intrauterine repair of fetal MMC improves the neurological prognosis compared with postnatal MMC repair (Adzick et al., 2011). However, the MOMS trial also proved that severe complications such as preterm birth, premature rupture of the membrane, and placental abruption and uterine wall dehiscence at the repair site increased due to a large incision of the uterine wall.

Coverage of a skin defect by a three-dimensional (3D) skin enables fetal intervention to be not only less invasive but also completed by an earlier term of pregnancy because autologous cultured skin transplantation enhances wound healing, as occurs in autologous skin transplantation for patients with severe burns (Pham et al., 2007). Moreover, 3D skin may grow in harmony with the fetal growth during all pregnancy periods. However, it is difficult to provide available autologous skin grafts for a fetus with MMC. Furthermore, the strong demand far outstrips the current supply of skin graft. Therefore, we hypothesize that induced pluripotent stem cells (iPSCs) can be an ideal material for autologous skin transplantation. The first clinical therapy using human iPSCs treated a Japanese woman with macular degeneration (Sivan et al., 2016). A great deal of research is currently in progress to facilitate therapy for patients with intractable diseases, such as Parkinson's disease and spinal cord injury (Cherry and Daley, 2012, Cherry and Daley, 2013). iPSCs can provide therapeutic potential for tissue repair and regeneration in combination with in vitro differentiation. The use of iPSCs in clinical application has largely been welcomed by society because the use of these cells avoids substantial ethical concerns and immune rejection.

In the present study, we generated human iPSCs from amniotic fluid cells (AFCs) using feeder-free, xeno-free, and integration-free systems. To verify that iPSCs could be generated from fetuses with severe fetal disease, we chose two major fetal diseases, twin-twin transfusion syndrome (TTTS) and trisomy 21 (T21): TTTS is one of the most serious complications of monochorionic multiple gestations, for which fetal intervention is most frequently performed, and trisomy 21 is the most common chromosome abnormality among live-born infants. To apply iPSC-based cell therapy during earlier gestational age, we established an effective protocol for differentiation of iPSCs into keratinocytes by the addition of Y-27632 and epidermal growth factor (EGF). We successfully established a surgical approach in the fetal rat model of MMC using reconstructed 3D skin with iPSC-derived keratinocytes (iPSC-KCs) and investigated the effect of transplantation of 3D skin.

## Results

### Derivation and Characterization of AFCs in Patients with Polyhydramnion

**Figure 1:**
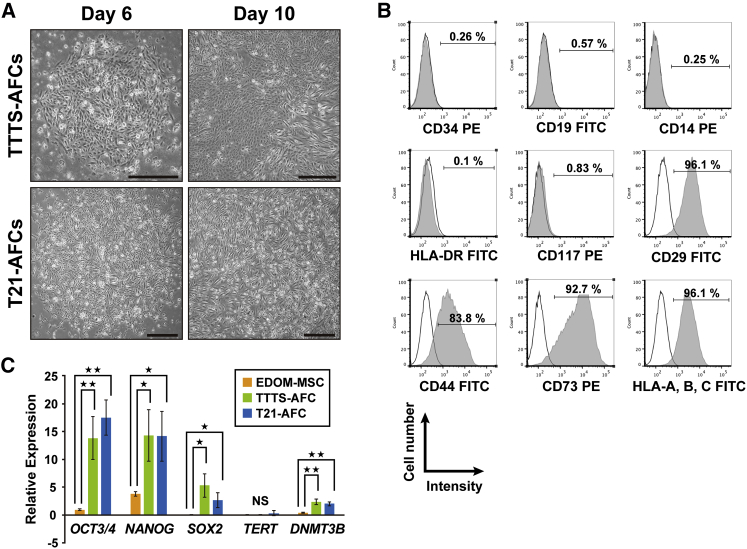
Characterization of Human Amniotic Fluid Cells Derived from Patients with Down Syndrome and Twin-Twin Transfusion Syndrome-Associated Polyhydramnion (A) Phase-contrast microscopic analysis for cell morphology in AFCs from patients with TTTS (TTTS-AFCs) and Down syndrome (T21-AFCs). Mesenchymal stem cell-like colonies appeared on the sixth day of culturing and tenth day of culturing. Scale bar, 500 μm. (B) Flow-cytometric analysis for CD29, CD44, CD73, CD117, CD14, CD19, CD34, human leukocyte antigen (HLA)-ABC, and HLA-DR. Isotype controls are shown in each panel. (C) Quantitative RT-PCR analysis for expression of *OCT3*/*4*, *NANOG*, *SOX2*, *TERT*, and *DNMT3B*. Values are shown as mean ± SD from three independent experiments. ^∗^p < 0.05, ^∗∗^p < 0.01.

To examine whether iPSCs could be efficiently generated from human AFCs derived from patients with the serious disease and coexisting polyhydramnion, we focused on two fetal diseases, namely TTTS and Down syndrome. Human AFCs were isolated from patients with TTTS (TTTS-AFC) and Down syndrome (T21-AFC). Amniotic fluid (AF) was obtained through amniocentesis under sterile conditions during amnioreduction for therapy of polyhydramnion. The average number of viable cells was 0.356 × 10^6^ ± 0.227 × 10^6^/mL (mean ± SD). The AFCs showed heterogeneous morphologies and reached confluence by 10 days after cell seeding (Figure 1A). Flow cytometry at passage 3 revealed that CD29, CD44, CD73, and human leukocyte antigen (HLA)-ABC (major histocompatibility complex [MHC] class I) were strongly positive; CD117 was rarely positive (0.8%), whereas CD14, CD19, CD34, HLA-DR, DP, and DQ (MHC class II) were negative (Figure 1B). Real-time qPCR analysis revealed trace amounts of *OCT3*/*4*, *NANOG*, and *SOX2*, compared with endometrial-derived mesenchymal stem cells (EDOM-MSCs) (Figure 1C). These results indicate that AFCs derived from polyhydramnion have a population similar to that of a normal volume of AF (De Coppi et al., 2007, Li et al., 2009, Tsai et al., 2004, You et al., 2008).

### Generation of Human iPSCs from AFCs of Patients with TTTS and Down Syndrome

**Figure 2:**
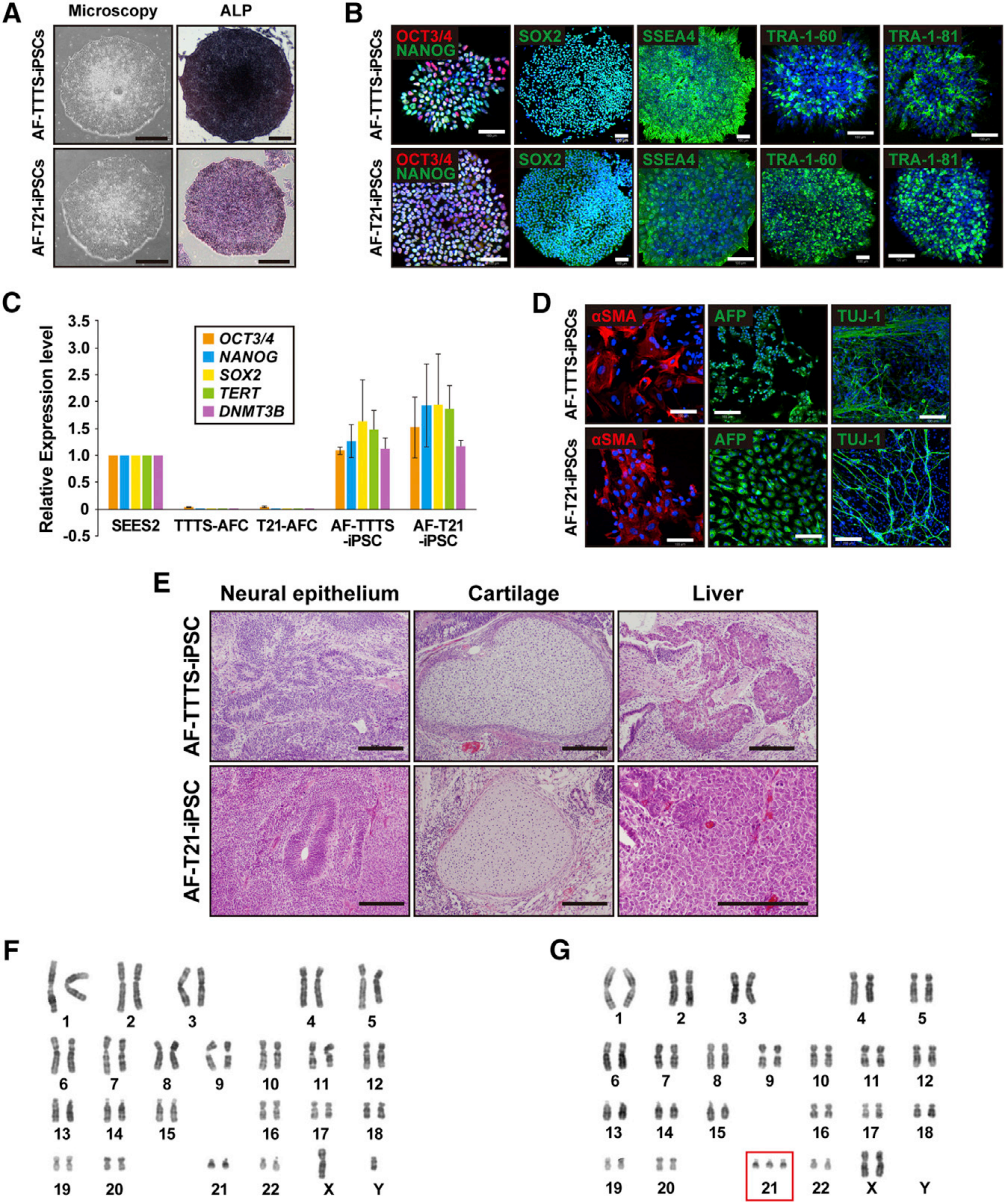
Generation of iPSCs from Patients with Down Syndrome T21 and Twin-Twin Transfusion Syndrome (A) Phase-contrast microscopic view of AF-TTTS-iPSC and AF-T21-iPSCs with embryonic stem cell (ESC)-like morphology growing on a feeder-free vitronectin surface (left panels). AF-TTTS-iPSCs and AF-T21-iPSCs are positive for alkaline phosphatase staining (ALP) (right panels). Scale bars, 500 μm. (B) Immunocytochemical analysis of stem cell markers, i.e., OCT3/4, NANOG, SOX2, SSEA-4, TRA-1-60, and TRA-1-81 in AF-TTTS-iPSC and AF-T21-iPSC colonies. AF-TTTS-iPSCs and AF-T21-iPSCs expressed these pluripotent markers. Nuclei were stained with DAPI (blue). Scale bars, 100 μm. (C) Real-time qPCR analysis of the endogenous expression levels of *OCT3*/*4*, *NANOG*, *SOX2*, *TERT*, and *DNMT3B* in AF-TTTS-iPSCs and AF-T21-iPSCs. The expression levels of these stem cell markers in AF-TTTS-iPSCs and AF-T21-iPSCs are comparable with those in human ESCs (SEES2). Values are shown as mean ± SD from three independent experiments. (D) In vitro differentiation of AF-TTTS-iPSCs and AF-T21-iPSCs into three germ layers. After EB formation, iPSCs were stained with antibodies to α-smooth muscle actin (αSMA) (a mesodermal marker), α-fetoprotein (AFP) (an endodermal marker), and βIII-tubulin (TUJ-1) (an ectodermal marker). Scale bars, 100 μm. (E) In vivo differentiation of AF-TTTS-iPSCs and AF-T21-iPSCs into three germ layers. Teratomas were harvested 6–8 weeks after subcutaneous injection of iPSCs into nude mice. Various tissues, such as neural epithelium (ectodermal), cartilage (mesoderm), and liver (endoderm), were found. Scale bars, 200 μm. (F) Karyotypic analysis in AF-TTTS-iPSCs. AF-TTTS-iPSCs had normal karyotypes (46, XY). (G) Karyotypic analysis in AF-T21-iPSCs. AF-T21-iPSCs had typical trisomy karyotypes (47, XX, +21). See also Figure S1.

AFCs at passages 3–4 were transfected with episomal vectors carrying six reprogramming factors (*L-MYC*, *KLF4*, *OCT4*, *SOX2*, *LIN28*, and short hairpin RNA for *P53*) by electroporation. We observed the appearance of human embryonic stem cell (ESC)-like colonies 30–50 days after electroporation. Colonies with human ESC-like morphology expanded and grew as flat colonies with large nucleocytoplasmic ratios with a high level of alkaline phosphatase activity (Figure 2A). Neither cessation of cell proliferation, such as senescence, nor apoptosis/cell death was detected during cultivation through 70 passages. iPSCs from TTTS-AFCs and T21-AFCs were designated as AF-TTTS-iPSCs and AF-T21-iPSCs, respectively. The reprogramming efficiency was 0.1% and 0.3% in AF-TTTS-iPSCs and AF-T21-iPSCs, respectively.

### Characterization of AF-T21-iPSCs and AF-TTTS-iPSCs

Both AF-TTTS-iPSCs and AF-T21-iPSCs expressed multiple pluripotency markers, including nuclear transcription factors OCT3/4, NANOG, and SOX2, as well as surface antigen stage-specific embryonic antigen 4 (SSEA-4) and tumor-related antigen (TRA)-1-60 and TRA-1-81 (Figure 2B). Real-time qPCR analysis showed that endogenous pluripotency marker genes, including *OCT3*/*4*, *SOX2*, *NANOG*, *telomerase reverse transcriptase* (*TERT*), and *DNA methyltransferase 3 beta* (*DNMT3B*), were activated in human iPSCs to a similar extent as those in control human ESCs (SEES2) (Figure 2C), and the transgenes were fully silenced in AF-T21-iPSCs and AF-TTTS-iPSCs. We next examined the ability for in vitro differentiation by examining the expression of germ layer-specific markers in the embryoid body (EB) formation. AF-T21-iPSCs and AF-TTTS-iPSCs were capable of differentiating into all three germ layers in vitro (Figure 2D). To examine pluripotency of iPSC clones, we formed teratomas by implantation of AF-T21-iPSCs and AF-TTTS-iPSCs at the subcutaneous tissue of immunodeficient nude mice. Three independent AF-T21-iPSCs and AF-TTTS-iPSCs clones induced teratomas within 6–8 weeks after implantation. Histological analysis of paraffin-embedded sections demonstrated that all three primary germ layers were generated as shown in Figure 2E. Thus, all AF-T21-iPSCs and AF-TTTS-iPSCs clones examined had potential for multilineage differentiation in vivo. Although teratoma derived from AF-T21-iPSCs showed the presence of neuroblastoma-like tissue (Figure S1A) and liver tissue with a vacuolar structure (Figure S1B), Down syndrome-specific findings were not detected. Karyotypic analyses revealed that the AF-TTTS-iPSCs clones had normal karyotypes (Figure 2F), whereas the AF-T21-iPSCs clones had trisomy 21 (Figure 2G). Analysis of 16 patterns of the short tandem repeat (STR) site revealed that all STR sites of AF-TTTS-iPSCs and AF-T21-iPSCs matched those of their parental TTTS-AFC and T21-AFC (Figure S1C).

### Whole-Exome Analysis of AF-T21-iPSCs

The whole-exome analysis, wherein our sample was compared with the GRCh37 reference sequence, detected a heterozygous single-nucleotide variant (SNV) in the *CRELD1* focus. The C-to-G substitution (rs2302787), which results in a Pro-to-Arg alteration, was situated in exon 4. Several mutations of *CRELD1* have been reported to contribute to occurrence of cardiac atrioventricular septal defects in Down syndrome (Maslen et al., 2006). To find out whether the alteration is deleterious, we employed SIFT and Polyphen2. The former makes influence from similarity of amino acid sequences and gives scores close to zero when a variant is damaging, whereas the latter predicts effects of not only sequences but also 3D structures and provides scores close to 1.0 when a variant is intolerant. The scores for the variants were 0 and 0.999, respectively, suggesting a notable variant. Although its global allele frequency was 1.0%, a higher frequency of 4.5% was documented for the Japanese population in the 1,000 Genomes project.

### Generation and Characteristics of iPSC-Derived Keratinocytes

**Figure 3:**
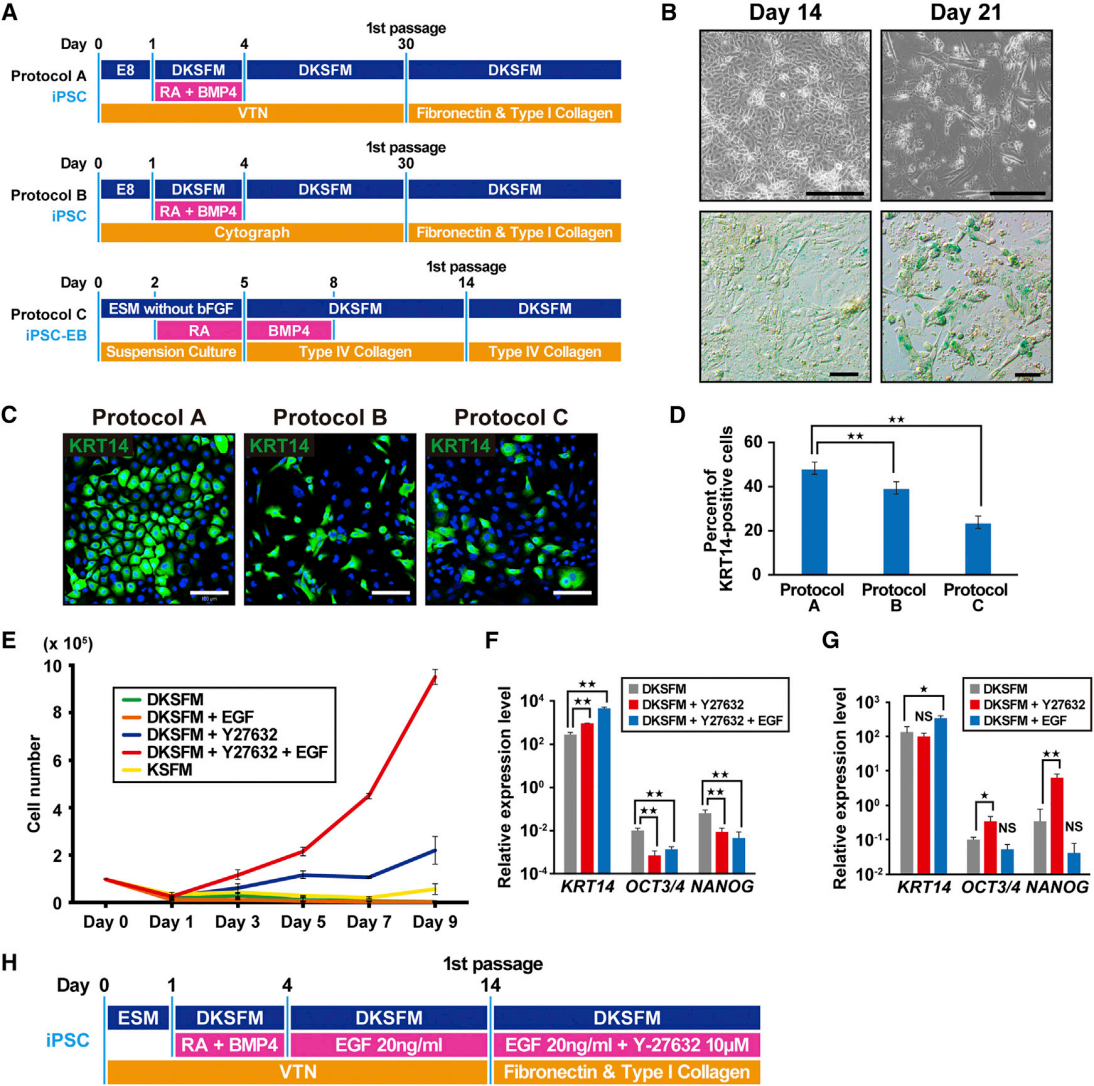
Establishment of Differentiation Protocol of iPSCs into the Lineage of Keratinocytes (A) Schematic of the three differentiation protocols for generation of keratinocytes from iPSCs. Protocols A and B differed in the coating agents. Protocol C was performed via embryoid body (EB) formation (iPSC-EB). DKSFM, defined keratinocyte serum-free medium; RA, retinoic acid; BMP4, bone morphogenetic protein 4; VTN, vitronectin; E8, Essential 8 medium; ESM, ESC medium. (B) β-Galactosidase staining of iPSC-KCs at the indicated time points (days 14 and 21). Cell senescence was observed at day 21 of the induction. Scale bars, 100 μm. (C) iPSC-derived keratinocytes with different methods (protocols A, B, and C) at passage 2 were immunocytochemically stained with anti- KRT14 antibody. Homogeneous keratinocyte-like cells were stained in protocol A. Scale bars, 100 μm. (D) The number of cells positive for KRT14. The number of KRT14-positive cells were highest in protocol A (48.08%), compared with protocol B (39.24%) and protocol C (23.62%). Data are shown as mean ± SD of the cell number from three independent experiments. KRT14, keratin 14. ^∗∗^p < 0.01. (E) The growth rate of iPSC-derived keratinocytes with different treatment. A combination of Y-27632- and EGF accelerated cell growth. EGF, epidermal growth factor; DKSFM, defined keratinocyte serum-free medium; KSFM, keratinocyte serum-free medium. (F) Real-time qPCR analysis of *KRT14* and stem cell markers, i.e., *OCT3*/*4* and *NANOG*, in iPSC-KCs at passage 2. *KRT14* expression increased by the addition of epidermal growth factor (EGF). Data shown are mean ± SD of the expressions from three independent experiments. ^∗∗^p < 0.01. (G) Real-time qPCR analysis of *KRT14* and stem cell markers in iPSC-KCs at passage 0. Gene expression levels of *OCT3*/*4* and *NANOG* increased in the presence of Y-27632, whereas the *KRT14* expression level remained unchanged by the treatment of Y-27632. Data are presented as mean ± SD from three independent experiments. ^∗^p < 0.05, ^∗∗^p < 0.01. NS, not significant. (H) Schematic of the final protocol design for differentiation of iPSCs into keratinocytes. iPSC-KC, iPSC-derived keratinocytes; KSFM, keratinocyte serum-free medium; DKSFM, defined keratinocyte serum-free medium; RA, retinoic acid; BMP4, bone morphogenetic protein 4. See also Figure S2.

We first attempted to generate iPSC-derived keratinocytes (iPSC-KC) based on the prior differentiation protocol (Bilousova et al., 2011, Guenou et al., 2009, Itoh et al., 2011, Metallo et al., 2008, Veraitch et al., 2013) using retinoic acid (RA) to promote ectodermal fate and BMP4 to block neural fate. To define the effective differentiation protocol, we compared differentiation efficiencies among three different protocols including direct differentiation using a VTN-coated dish (protocol A), CytoGraph-coated dish (protocol B), and the EB method (protocol C) (Figure 3A). Protocols A and B differed with respect to coating agent. In protocol A, we modified the previously reported protocol (Itoh et al., 2011) by replacing Matrigel with a human recombinant protein using VTN. During direct differentiation (protocols A and B) cell senescence was observed at day 30, and these cells could not proliferate after the first passage. The number of keratinocyte-like cells decreased after 17 days and β-galactosidase staining revealed that cellular senescence was observed over 17 days, resulting in an exacerbated cellular state (Figure 3B). Therefore, the first passage was performed at 14–17 days in protocols A and B, respectively.

### Effect of Y-27632 on iPSC-KCs

Although we detected keratinocyte-like cells during passage 1 of protocols A, B, and C, we examined additional factors to obtain a sufficient number of iPSC-KC and found that Y-27632 was critical. Keratinocytes derived from iPSCs grown in the presence of Y27632 showed improved cell growth. The comparison of immunostaining of KERATIN 14 (KRT14) at passage 2 with protocols A, B, and C revealed that the percentage of KRT14-positive cells reached 48.08%, 39.24%, and 23.62% in protocol A, B, and C, respectively, indicating that protocol A is most suitable for iPSC-KC proliferation (Figures 3C and 3D). Therefore, during subsequent experiments differentiation of iPSCs into keratinocytes was performed by protocol A.

### Effect of EGF on iPSC-KCs

iPSC-KCs treated with Y-27632 showed improved cell growth; however, cell proliferation remained insufficient and required more than 2 weeks to obtain a sufficient cell number. To promote cell proliferation with a high expression level of epithelial markers, we examined iPSC-KCs to investigate the effect of EGF. iPSC-KCs treated with EGF and Y-27632 for 9 days after the first passage (starting cell number was 1 × 10^5^/well) showed markedly improved cell growth compared with the cells treated with Y-27632, whereas the cell number of EGF-treated iPSC-KCs without treatment of Y-27632 did not proliferate at all, indicating that the combination of EGF and Y-27632 is important for the cell growth of iPSC-KCs (Figure 3E). Although iPSC-KCs grown in keratinocyte serum-free medium (KSFM) showed a higher cell growth than that grown in defined KSFM (DKSFM) (Figure 3E), we used DKSFM for subsequent experiments as it is chemically defined and optimized for growth and expansion of human keratinocytes without the potential contamination derived from animal serum. We further analyzed gene expression levels of *KRT14* in iPSC-KC at passage 1 and found that iPSC-KCs treated with EGF and Y-27632 expressed *KRT14* at a higher level than those treated with Y-27632 alone (Figure 3F). Flow-cytometric analysis also showed that the KRT14-positive population increased in cell number and intensity by addition of Y-27632 and EGF (Figure S2A). Treatment with EGF from day 4 to day 14 resulted in a marginal yet significantly higher expression of *KRT14* (Figure 3G). Y-27632 was not included for days 4–14 because Y-27632-treated iPSC-KCs before the first passage showed a higher expression of *OCT3*/*4* and *NANOG* (Figure 3G). SSEA4-positive undifferentiated iPSCs were successfully removed by treatment of EGF and Y-27632 after the first passage, as confirmed by flow-cytometric analysis (Figure S2B). As for the growth medium, DKSFM was used throughout the differentiation process (Figure S2C). We additionally compared the cell proliferation of iPSC-KCs at passage 1 on the matrix combinations with type I collagen, type IV collagen, and fibronectin. The combination of “type I collagen and fibronectin” was the most suitable for the cell proliferation of iPSC-KCs (Figure S2D). Taken together, we concluded that differentiation efficiency of iPSC-KCs was most effective in protocol A using DKSFM-supplemented EGF and Y-27632, as shown in Figure 3H.

### Characterization of Keratinocytes Derived from iPSCs

**Figure 4:**
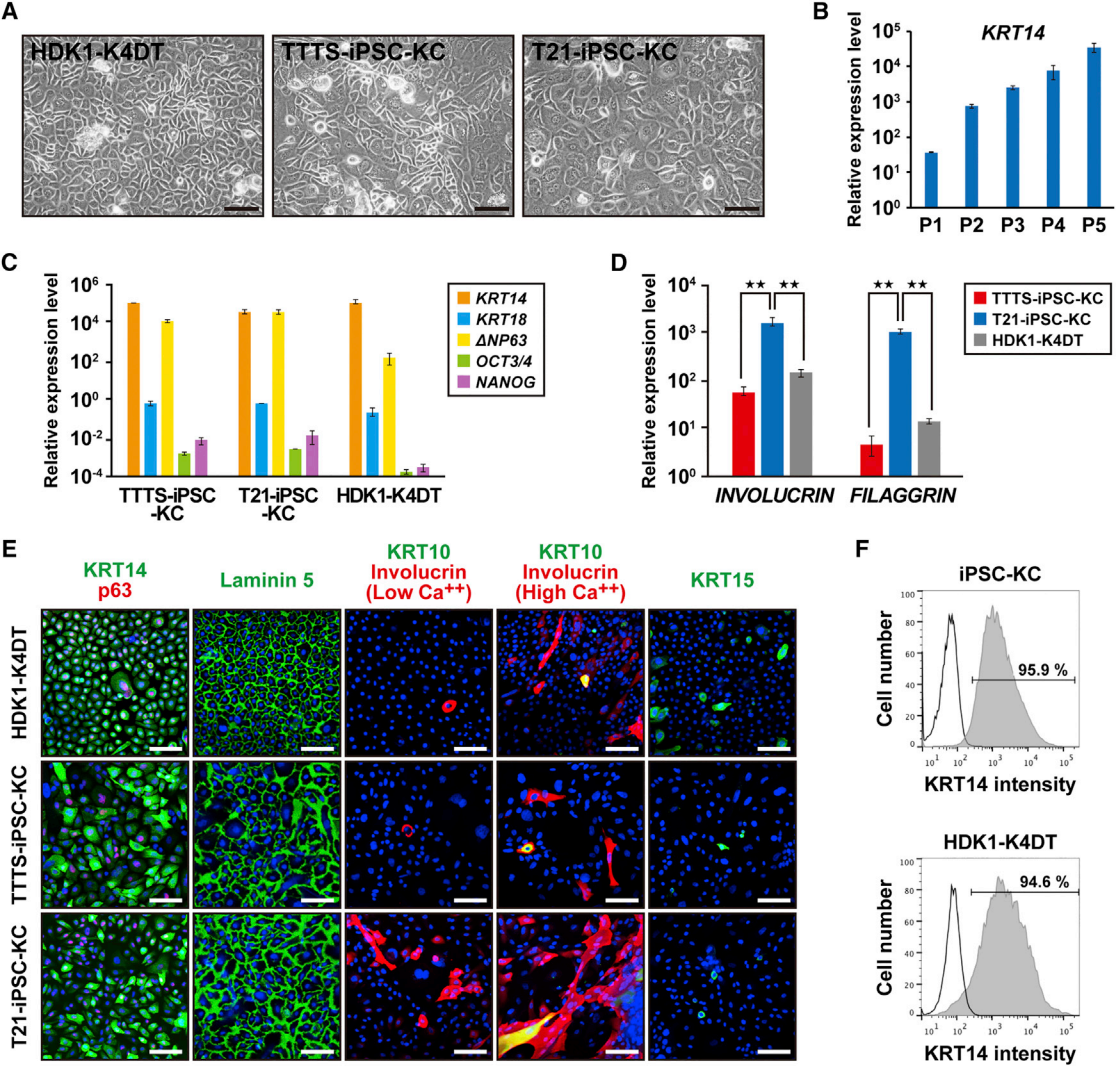
Characterization of a Homogeneous Population of Keratinocytes Derived from Induced Pluripotent Stem Cells (A) Phase-contrast microscopic analysis of human dermal keratinocytes (HDK1-K4DT) and keratinocytes derived from AF-T21-iPSCs (T21-iPSC-KC) and AF-TTTS-iPSCs (TTTS-iPSC-KC). Both iPSC-derived keratinocytes showed human keratinocyte-like morphology. Image of “T21-iPSC-KC” is identical to “iPSC-KC at passage 3–4” in Figure S3B. Scale bars, 500 μm. (B) Real-time qPCR analysis of *KRT14* at each passage. Values are shown as means ± SD from three independent experiments. (C) Real-time qPCR analysis of epithelial markers, i.e., *KRT14*, *KRT18*, and *ΔNP63*, and stem cell markers, i.e., *OCT3*/*4* and *NANOG*, in human iPSC-KCs at passage 4. The epithelial marker expression levels in iPSC-KCs were similar to those of HDK1-K4DT. The expression levels of each gene in the parental iPSCs were regarded as equal to 1 (10^0^). Values are shown as means ± SD from three independent experiments. (D) Real-time qPCR analysis of terminal differentiation markers, i.e., *INVOLUCRIN* and *FILAGGRIN*. T21-iPSC-KCs showed higher expression levels of *INVOLUCRIN* and *FILAGGRIN* than did TTTS-iPSC-KCs. The expression levels of each gene in the parental iPSCs were regarded as equal to 1 (10^0^). Values are shown as means ± SD from three independent experiments. ^∗∗^p < 0.01. (E) Immunofluorescence of epithelial markers, i.e., KRT14, p63, laminin 5, involucrin, KRT10, and KRT15. Under the high-calcium condition, iPSC-KCs expressed involucrin and KRT10 at a higher frequency, indicating accelerated epidermal differentiation. Scale bars, 100 μm. (F) Flow-cytometric analysis of KRT14 in iPSC-KCs at passage 2. Isotype controls are included in each panel. See also Figures S3 and S4.

In our protocol, overall induction periods were further reduced compared with previously reported protocols (Guenou et al., 2009, Itoh et al., 2011, Itoh et al., 2013), and iPSC-KCs were successfully increased and could be expanded for more than five passages at least. Interestingly, in terms of proliferative and differentiation abilities, both Y-27632 and EGF were required for the generation of iPSC-KC. Y-27632 had a greater effect on the proliferative ability, and EGF affected more the terminal differentiation. Keratinocytes derived from AF-TTTS-iPSCs (TTTS-iPSC-KCs) and AF-T21-iPSCs (T21-iPSC-KCs) showed keratinocyte-like morphology at passage 4, like HDK1-K4DT (Figure 4A). *KRT14* gradually increased for each passage (Figure 4B). Transcriptional analysis by real-time qPCR showed that the gene expression levels of *KRT14*, *KRT18*, *p63*, *OCT3*/*4*, and *NANOG* of TTTS-iPSC-KCs and T21-iPSC-KCs at passage 4 reached those in HDK1-K4DT (Figure 4C). Interestingly, T21-iPSC-KCs showed higher expression of terminal differentiation markers such as *INVOLUCRIN* and *FILAGGRIN* than TTTS-iPSC-KCs (Figure 4D). This result is consistent with the clinical features of hyperkeratosis that are frequently observed in patients with Down syndrome. Immunostaining revealed the expression of KRT14, p63, and laminin 5 in both TTTS-iPSC-KCs and T21-iPSC-KCs (Figure 4E). Moreover, KRT10 and involucrin, markers of differentiated keratinocytes of the suprabasal layer, were induced under a high-calcium condition. As expected, the number of involucrin-positive T21-iPSC-KCs increased under a low calcium condition. KRT15, a marker of epithelial stem cells in the hair follicle, was rarely detected (Figure 4E). KRT15 was not detected throughout the differentiation process. The KRT14-positive cell population in EGF and Y-27632-treated iPSC-KC at passage 2 reached to 95.9%, as shown by flow-cytometric analysis (Figure 4F).

### Passage-Dependent Epidermal Differentiation of iPSC-KCs for 3D Skin

**Figure 5:**
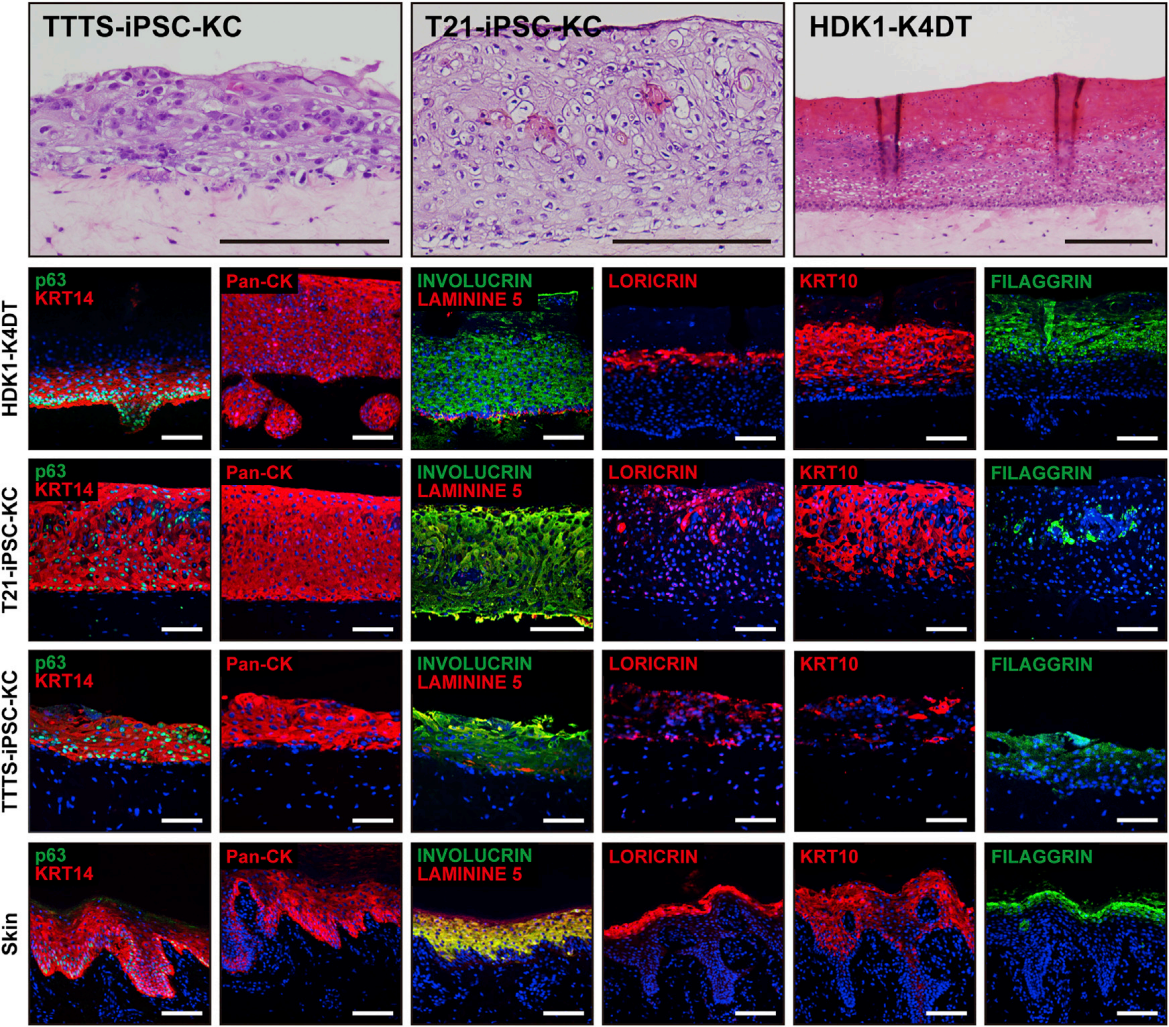
Three-Dimensional Cultured Skin Equivalents Using iPSC-KCs (Upper panels) Histology of 3D skin with T21-iPSC-KCs, TTTS-iPSC-KCs, or HDK1-K4DT. (Lower panels) Immunohistochemical analysis with antibodies to KRT14, p63, Pan-CK, involucrin, laminin 5, loricrin, KRT10, and filaggrin. The multilayered epidermis expressed KRT14, involucrin, laminin 5, Pan-CK, loricrin, KRT10, and filaggrin in artificial skin, indicating that iPSC-KCs terminally differentiate in these skin equivalents. The expression patterns of these markers in intact skin are shown for reference. Scale bars, 100 μm. See also Figure S5.

iPSC-KCs reduced proliferative ability as each passage progressed; however, their terminal differentiation ability increased as each passage progressed (Figures 4B and S3A). Cell morphology of iPSC-KCs appeared spindle-like during early passages and became similar to that of human keratinocytes as the passages progressed (Figure S3B). However, a vacuolar degeneration-like structure was observed at passage 5, and the cells ceased to proliferate (Figure S3B). For the successful construction of 3D skin with iPSC-KCs, we investigated proper passage number of iPSC-KCs with both sufficient expression levels of KRT14 and the ability of cells to grow. iPSC-KCs at passage 0 constructed epidermis that was negative for Ki67, KRT14, p63, and pan-cytokeratin (Pan-CK) (Figure S4). The 3D skin with iPSC-KCs at passage 1 revealed immature epithelial-like tissue that expressed pan-CK and Ki67 but not other epithelial markers, including KRT14 and P63 (Figure S4). The 3D skin with iPSC-KCs at later passages (more than passage 4) showed mature keratinocytes with the prickle cell layer that were strongly positive for KRT14, Pan-CK, and involucrin, and weakly positive for Ki67 (Figure S4). The 3D skin with iPSC-KCs increased the terminal differentiation markers and morphologically matured as the passage numbers of iPSC-KCs increased. Finally, we successfully identified the most suitable condition to generate a multilayered epidermis with iPSC-KCs at passage 3. The 3D skin with iPSC-KCs at passage 3 after 14 days of air-liquid cultivation had a multilayered epidermis with the KRT14 in the basal compartment and laminin 5 at the dermal-epidermal junction (Figure 5). Moreover, KRT10 was detected in the suprabasal layer, and loricrin and filaggrin, late markers of epidermal differentiation, were also observed in the upper layers of the epidermis. The expression patterns of epithelial markers in the 3D skin resembled those in intact human skin. These data suggest that AF-TTTS-iPSCs and AF-T21-iPSCs can be differentiated into fully functional keratinocytes for artificial 3D skin. This success may be attributed to the favorable balance of proliferation and differentiation capacities in iPSC-KCs.

### Transplantation of 3D Skin into MMC Fetal Rats

**Figure 6:**
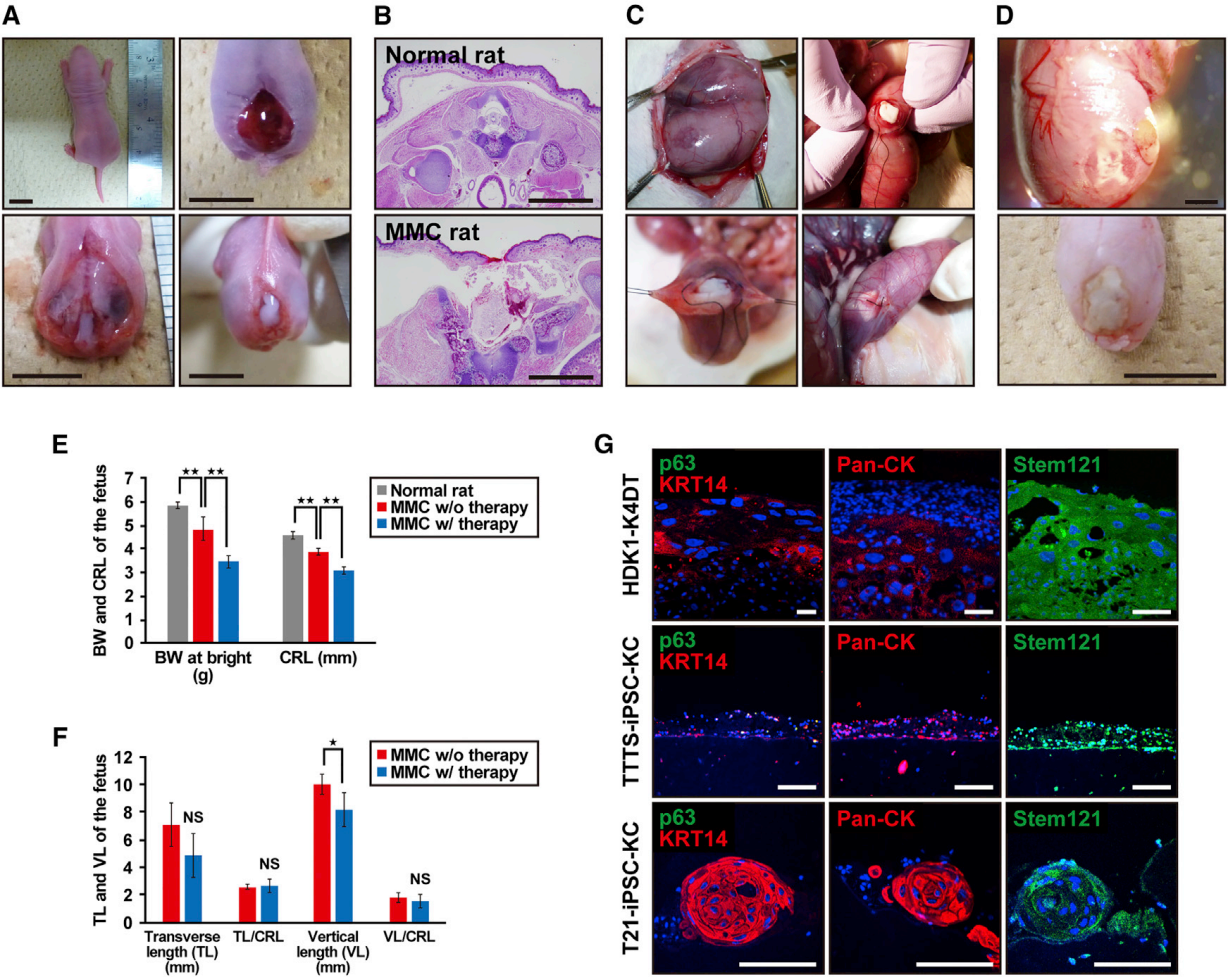
The Rat Model of Myelomeningocele with Application of Artificial Skin (A) Gross pathology images of the MMC lesion site at birth in a normal rat (upper left panel) and MMC rat (upper right and lower panels). MMC rat shows defects in skin (upper right) and spine (lower left), and exposure of spinal cord (lower right). Scale bars, 1 cm. (B) Cross-sectional images of spinal cord at lumbar levels of a normal rat and MMC rat. Scale bars, 2 mm. (C) Gross view of the intrauterine transplantation of artificial skin. Representative images of major steps of the MMC repair process in the fetal rat model. An MMC defect site was identified through the uterus (upper left panel). A small incision of the uterus was made just above the defect site, following which artificial skin was transplanted (upper right panel). Finally, the uterine wall was closed over the defect (lower panels). (D) Gross views of neonatal rat with myelomeningocele (MMC) at birth (E22). Representative photograph in incomplete closure (upper panel) and complete closure (lower panel) after artificial skin application at day 20. Scale bars, 2 mm (upper panel) and 1 cm (lower panel). (E) Birth weight (BW) and craniocaudal length (CRL) of normal neonatal and neonatal MMC rats with or without fetal therapy. MMC rats in the fetal therapy group (n = 3) were born with smaller BW and CRL compared with normal rats (n = 14) or nontreated MMC rats (n = 6). ^∗∗^p < 0.01. (F) Comparison of MMC defect size between neonatal MMC rats with fetal treatment (n = 3) and rats without therapy (n = 6). There were no significant differences in the MMC size by fetal therapy when adjusted for the CRL. TL, transverse length; VL, vertical length. ^∗^p < 0.05; NS, not significant. (G) Expression profile of epidermal markers and short-term in vivo effect on regeneration of rat skin defect in artificial skin. Transverse section through the myelomeningocele defect 2 days after transplantation of artificial skin with iPSC-KC epidermis. The expressions of epidermal markers, i.e., KRT14, p63, pan-cytokeratin (Pan-CK), and stem121, were analyzed in artificial skin with HDK1-K4DT, TTTS-iPSC-KCs, or T21-iPSC-KCs. Scale bars, 100 μm.

A total of 97 fetuses were viable after cesarean section. MMC was present in 83.5% (81 of 97) of fetuses exposed to RA. The MMC rats showed a defect in the skin and spine and exposure of the spinal cord (Figure 6A). Cross-sectional analysis confirmed the MMC defects and showed the degenerated spinal cord in rats exposed to RA (Figure 6B). In total, 20 fetal rats were operated on at day 20 of gestation. The 3D skin with iPSC-KCs was transplanted into fetuses across a small incision of the uterine wall following closure of the incision site (Figure 6C). Twelve neonatal rats had a complete or partial skin defect coverage with 3D skin (complete coverage: 4 of 20 [20%], incomplete coverage: 8 of 20 [40%]), virtually isolating the spinal cord from direct exposure to the amniotic cavity (Figure 6D). Birth weight and crown-rump length (CRL) were significantly lower in the fetal therapy group than in normal rats and nontreated MMC rats (Figure 6E). Although the transverse length (TL) and vertical length (VL) of the MMC defect size were slightly shorter in the fetal therapy group than in fetuses without therapy, there were no significant differences when the TL and VL were adjusted for overall fetal CRL (Figure 6F).

### Histological Evaluation of 3D Skin at Transplantation Site

Immunohistochemistry of the transplanted artificial skin with iPSC-KCs and HDK1-K4DT demonstrated that the expression of epithelial markers, such as KRT14 and p63, was weak, but obviously remained after birth (Figure 6G). Transplanted 3D skin was confirmed using human-specific antigen cytoplasmic protein detected with “stem121.” Moreover, epidermal ingrowth was detected at the edge of the epidermis defect in MMC rats, and the epidermis appeared to be elongated underneath the transplantation site (Figure S5). iPSC-KCs did not form any tumors, i.e., teratomas or malignant lesions, implying that iPSC-KCs are expected to be safe from the viewpoint of tumorigenicity.

## Discussion

This study is designed to obtain preclinical proof of concept for fetal therapy to patients with MMC using autologous iPSCs. Pregnant women with polyhydramnion need to receive amnioreduction therapy; i.e., 1,000–2,000 mL of AF is usually aspirated for preventing threatened premature delivery and respiratory discomfort. A large number of AFCs can be obtained from 20 mL of AF by amnioreduction. The sufficient number of AFCs with their prominent proliferation capability leads to successful iPSC generation from patients with lethal disorders, such as TTTS.

### Challenge of an iPSC Therapy to Diseased Fetus

In this study, we hypothesize that AFC-derived iPSCs can be used for diseased fetus therapy. The differentiation protocol developed in this study enables earlier induction of keratinocytes from iPSCs than has been previously reported. AFCs can be prepared at 15 weeks of gestation. It takes 2 or 3 weeks to establish iPSCs, and sufficient numbers of iPSCs as a starting material can thus be obtained by 23 weeks of gestation or earlier. Epidermis and 3D skin can be formed by 28 weeks of gestation. In total, generation of 3D skin takes approximately 14 weeks from AF preparation to generate 3D skin. Fetal surgeries of MMC have been performed at up to 29 weeks of gestation at present (Adzick et al., 2011, Degenhardt et al., 2014, Pedreira et al., 2014). For infants with MMC, early term delivery (at approximately 37 weeks of gestation) is preferable to reduce risks of infant complications, including respiratory distress syndrome, necrotizing enterocolitis, cerebral hemorrhage, and infant death. The therapeutic effect of 3D skin transplantation is therefore evident if 3D skin implantation is performed at 28–29 weeks of gestation. 3D skin coverage is supposed to prevent chemical-induced injury to the spinal cord caused by AF and persistent effusion of cerebrospinal fluid, leading to the prevention of the progression of Chiari malformation and hydrocephalus. It is also noteworthy that the engrafted 3D skin regenerates with the growth of the fetus and accelerates skin coverage throughout the pregnancy period.

### Fetal Therapy to a Patient with MMC with 3D Skin

Fetal therapy started with patients with TTTS, fetal anemia, and MMC (Adzick and Harrison, 1994, De Lia et al., 1990, Rodeck et al., 1981); however, a standard procedure has not yet been developed (Danzer and Johnson, 2014). Regarding MMC, laparoscopic surgery to suture skin defects is one of the fetal therapies so far in cases where skin defects are not large. Artificial skin needs to be developed, due to coverage of large skin defects, along with a surgical approach. For generation of artificial 3D skin, iPSCs can be efficiently generated from AFCs derived from polyhydramnion in integration-free, xeno-free, and serum-free conditions because AFCs derived from patients with polyhydramnion exhibited prominent proliferative capacity in vitro.

An important limitation of this rat model is the short gestation period of rats without the ability to analyze the longer-term effects of artificial skin in vivo. Further studies to analyze the longer-term effect, including regeneration of skin, tumorigenic potential, and neurological prognosis, are required in a large animal model. In the fetal therapy strategy, improvement in distal neurological function remains limited because of the failure to reverse neurological injury that occurred prior to the time of repair (Heffez et al., 1990). The transplantation of neural stem cells was reported to improve neurological outcome in an animal model of nerve injury, such as spinal cord injury (Biernaskie et al., 2007, Hu et al., 2010, Sieber-Blum, 2010, Wang et al., 2011). iPSC-derived neural crest stem cells integrate into the injured spinal cord in the fetal lamb model of MMC (Saadai et al., 2013). Additional cell types, such as neural stem cells/neural crest cells, to the artificial skin may be beneficial to a future fetal therapy.

In this study, we demonstrated a fetal MMC therapy that can be achieved by cellular therapy using AFC-derived iPSCs. Our fetal cell treatment is minimally invasive and therefore has the potential to become an effective treatment for MMC.

## Experimental Procedures

### Ethical Statement

The protocol for the use of human cells in the present study was approved by the Institutional Review Board of the National Research Institute for Child Health and Development of Japan and was in full compliance with the Ethical Guidelines for Clinical Studies (Ministry of Health, Labor, and Welfare).

### Human Cells

AF was obtained from fetuses with Down syndrome and TTTS, with both conditions associated with polyhydramnion. In the case of TTTS, AF was obtained at the time of fetoscopic laser surgery at gestational ages ranging from the 19th to 26th weeks. In the cases of Down syndrome, AF was obtained by amnioreduction at 29 weeks of gestation. Cells were incubated in 4 mL of AmnioMAX-II Complete Medium (Invitrogen, catalog number (#)11269-016). Cell clusters were emerged at 6–7 days after seeding. Nonadherent cells were discarded and the medium was changed every 2 days. When the cultures reached subconfluence, the cells were harvested with a trypsin-EDTA solution (Wako, #209-16941) and replated at a ratio 1:8 in a 60-mm dish.

### Generation of Feeder-Free iPSCs

AFCs were seeded at 6 × 10^5^/well on 6-well plates. Three episomal vectors encoding six factors (*L-MYC*, *KLF4*, *OCT4*, *SOX2*, *LIN28*, and short hairpin RNA for *P53*) (Addgene, #27077, 27078, 27079) were electroporated into the AFCs on day 0 as previously described (Okita et al., 2011). On day 5, transfected cells were passaged and seeded at 1.3 × 10^6^/dish onto vitronectin (VTN) (Life Technologies, #A14701SA)-coated 100-mm plates in Essential 8 (E8) medium (Life Technologies, #A14666SA). We observed the appearance of human embryonic stem cell (ESC)-like colonies 30–50 days after electroporation. iPSCs were maintained in E8 medium on VTN-coated dishes and passaged using 0.5 mM EDTA in PBS.

### RT-PCR

cDNA was synthesized from 1 μg of total RNA using Superscript III reverse transcriptase (Invitrogen, #18080-085) with oligo(dT) primer according to the manufacturer's instructions. Template cDNA was PCR amplified with the gene-specific primer sets (Table S1).

### Real-Time qPCR

RNA was extracted from cells using the RNeasy Plus Micro kit (Qiagen, #74004). An aliquot of total RNA was reverse transcribed using an oligo(dT) primer (Invitrogen, #18418-20). For the thermal cycle reactions, the cDNA template was amplified (Applied Biosystems Quantstudio 12K Flex Real-Time PCR System) with gene-specific primer sets using the Platinum qPCR SuperMix-UDG with ROX (Invitrogen, #11743-100) under the following reaction conditions: 40 cycles of PCR (95°C for 15 s and 60°C for 1 min) after an initial denaturation (95°C for 2 min). Fluorescence was monitored during every PCR cycle at the annealing step. mRNA levels were normalized using glyceraldehyde-3-phosphate dehydrogenase as a housekeeping gene.

### Immunocytochemical Analysis

Cells were fixed with 4% paraformaldehyde (PFA) in PBS for 10 min at 4°C. After washing with PBS and treatment with 0.1% Triton X-100 (Sigma-Aldrich, #T8787-100 ML) for 10 min at 4°C, cells were preincubated with 5% normal goat serum (Dako, #X0907) in PBS for 30 min at room temperature, following which they were reacted with primary antibodies in blocking buffer for 24 hr at 4°C. After washing with PBS, cells were incubated with fluorescently coupled secondary antibodies; anti-rabbit or anti-mouse immunoglobulin G (IgG) conjugated with Alexa 488 or 546 (1:1,000) in blocking buffer for 30 min at room temperature. The nuclei were stained with DAPI (Biotium, #40043). All images were captured using confocal microscopy (LSM 510 and LSM 510 META laser scanning microscope). Antibody information is provided in Table S2.

### Karyotypic Analysis

Karyotypic analysis was performed at the Nihon Gene Research Laboratories. Chromosome spreads were Giemsa banded and photographed. Twenty metaphase spreads were analyzed for each sample and karyotyped using a chromosome imaging analyzer system (Applied Spectral Imaging).

### Short Tandem Repeat Analysis

STR analysis was performed at BEX’s facility. Using the genomic DNA, 16 microsatellite markers were amplified by PCR with microsatellite-specific primers.

### Whole-Exome Sequencing

Approximately 2.0 μg of genomic DNA from each cell sample was sonicated to provide an average fragment size of 200–300 bp on a Covaris S220 instrument. After five cycles of PCR amplification, capture and library preparation were performed with Agilent SureSelect Human All Exon V5+ long intergenic noncoding RNA (50 Mb), followed by washing, elution, and additional 12-cycle PCR amplification to attach index adaptors. Enriched libraries were sequenced on an Illumina HiSeq 2500 operated in 100-bp paired-end mode. Image analyses and base calling on all lanes of data were performed using bcl2fastq 1.8.4 with default parameters.

### Read Mapping and Variant Analysis

Reads from the sample were first trimmed by removing adapters using cutadapt 1.7.1 and low-quality bases at ends using a custom script. They were then aligned to the hs37d5 sequence (hg19 and decoy sequences) using the Burrows-Wheeler Aligner 0.7.10. Mapped reads were converted from SAM to BAM using SAMtools 1.2 and processed by Picard 1.109 to eliminate PCR duplicate reads. The Genome Analysis Toolkit (GATK) 3.4 was then used to perform local realignment with known indel sites and map quality score recalibration to produce calibrated BAM files. Multi-sample callings for SNVs were made by GATK. The annotated variant call format files were then filtered using GATK with a stringent filter setting and custom scripts. Annotations of detected variants were made using ANNOVAR based on GRCh37. Genotypes of control samples were shuffled for each variant from a perspective of protection of personal information.

### Teratoma Formation

iPSCs (>1 × 10^7^) were subcutaneously inoculated into immunodeficient nude mice (BALB/cAJcl-*nu*/*nu*) (Crea). After 6–8 weeks, the resulting tumors were dissected.

### Fluorescence-Activated Cell Sorting Analysis

The expression of cell surface markers was analyzed by BD LSR Fortessa (BD Biosciences). Primary antibodies were incubated for 1 hr in PBS with 1% BSA. After washing with PBS, cells were incubated with fluorescently coupled secondary antibodies; anti-rabbit or anti-mouse IgG conjugated with Alexa 488 (1:1,000) for 30 min at room temperature.

### Protocol for Differentiating iPSCs into Keratinocytes

To induce differentiation, we subcultured small clumps of undifferentiated iPSCs onto a VTN-coated 10-mm dish in E8 medium on day 1 (protocol A). For comparison of differentiation protocols, single cells of iPSCs were subcultured onto a VTN-coated circle patterned CytoGraph (Dai Nippon Printing) dish in E8 medium-supplemented 10-μM Y-27632 (Wako, #251-00514) (protocol B) on day 1. iPSCs were then incubated in DKSFM (Invitrogen, #10744-019) supplemented with 1 μM all-*trans* RA (Wako, #182-01111) and 10 ng/mL bone morphogenetic protein 4 (BMP4) (R&D systems, #314-BP-010/CF) for 4 days. After 4 days, iPSCs were maintained in DKSFM supplemented with 20 ng/mL EGF (R&D systems, #236-EG-200) until 14 days and passaged onto a 0.03-mg/mL coating of type I collagen (Advanced Biomatrix, #5005-B) and 0.01 mg/mL fibronectin (Sigma-Aldrich, #F0895-1MG)-coated dish in DKSFM supplemented with 10 μM Y-27632 (Wako, #251-00514) and 20 ng/mL EGF. iPSC-derived keratinocytes were seeded at 3 × 10^4^ cells/cm^2^ and enriched by rapid adherence to fibronectin and type I collagen-coated dishes for 15–30 min at room temperature. Nonadherent cells were discarded and rapidly attached cells were cultured. The EB method (protocol C) was performed as described previously (Bilousova et al., 2011). EBs (n = 100) were formed from 5 × 10^4^ iPSCs on a Petri dish in embryonic stem cell medium without basic fibroblast growth factor (ESC-no-bFGF medium). After 2 days, 30 EBs were transferred to a new Petri dish with ESC-no-bFGF medium containing 1 μM RA for 3 days in suspension culture. EBs were then transferred onto a type IV (Sigma-Aldrich, #C7521-10MG)-coated 100-mm dish with ESC-no-bFGF medium containing 25 ng/mL BMP4. After 3 days the medium was switched to DKSFM, and the plated EBs were cultured for 6 days. EB remnants were removed by vacuum aspiration, and iPSC-derived keratinocytes were subcultured onto a type-IV-coated 100-mm dish and rapidly attached to a type-IV-coated dish for 15 min at room temperature in DKSFM.

### Generation of 3D Skin Equivalent

3D skin was generated according to a previously described protocol (Tsunenaga et al., 1994). Type I collagen (Koken, #IPC-50) and 1 × 10^6^ HFF2s were mixed and poured into an untreated 60-mm Petri dish while cooling and allowed to gel at 37°C for 1 hr to prepare the dermal equivalent. Contraction of the collagen gel was facilitated by pulling the gel from the surface of the Petri dish. The medium was changed every 2 or 3 days for 7 days. HDK1-K4DT or iPSC-derived keratinocytes were plated at 2 × 10^5^ or 1 × 10^6^ cells inside in a glass ring (10 mm diameter) on the surface of the contracted collagen gel, which was plated onto polyethylene terephthalate membranes (Corning, #35-3493). iPSC-derived keratinocytes were grown in DKSFM supplemented with 10 μM Y-27632 and 20 ng/mL EGF for 2 days, following which they were exposed to air in a 1:1 mixture of KSFM and DMEM plus 10% FBS, in which the Ca^2+^ concentration was adjusted to 1.8 mM. The medium was changed every 2 or 3 days. Multilayered 3D cultures of keratinocytes were obtained by days 14–21.

### Animal Preparation and RA Exposure

The procedure for creating MMC defects in fetal rats was based on the protocol described earlier (Danzer et al., 2005, Watanabe et al., 2010). In brief, time-dated Sprague-Dawley rats (Clea Japan or Sankyo Labo Service) were used. After being exposed to isoflurane (Wako, #099-065-71), anesthetized rats were fed 60 mg/kg all-*trans* RA (Wako, #182-01111) dissolved in olive oil (10 mg/mL) at embryonic day 10 (E10).

### Surgical Procedure

Fetal intervention of RA-exposed Sprague-Dawley rats was performed at E20. Pregnant rats were anesthetized by isoflurane, and an abdominal midline incision was made to expose the uterine horns. The MMC defect was confirmed through the uterus under direct vision, and a small incision of the uterine wall and amniotic membranes was made directly above the defected site. 3D skin was transplanted into the area of defected skin. Beriplast P Combi-Set (CSL Behring, #87799) was used for tissue adhesion. The hysterectomy site was closed by purse-string suture with 6-0 silk (Natsume Seisakusho, #B10-60). The uterus was returned to the abdomen and the abdominal incision site was closed by running suture. The fetuses were harvested by cesarean section at E22. Pregnant rats were then euthanized by cervical dislocation under anesthesia with isoflurane. The transplantation site was dissected and processed for histological and immunohistochemistry analysis. The primary antibody list is provided in Table S2. Appropriate Alexia 488- or Alexa 594-conjugated secondary antibody was used with DAPI nuclear counterstain. The operation protocols were accepted by the Laboratory Animal Care and the Use Committee (A2015-003-C01).

### Statistical Analysis

Each experiment was repeated at least three times, and the data are presented as the mean ± SD of the mean. Statistical significance was determined by Student's *t* test. p < 0.05 was considered statistically significant.

## Author Contributions

K.K., T.T., S.W., H.S., A.O., H.A., and A.U. designed experiments. K.K., J.K., Y.I., and Y.O. performed experiments. K.O. and S.T. analyzed data. T.K. contributed reagents, materials, and analysis tools. K.K., N.I., O.S., and A.U. discussed the data and manuscript. A.U. and M.S. wrote the manuscript.

## Supplemental Information

Document S1. Figures S1–S5 and Tables S1 and S2

Document S2. Article plus Supplemental Information
