# Supplementary material for: Fetal Therapy Model of Myelomeningocele with Three-Dimensional Skin Using Amniotic Fluid Cell-Derived Induced Pluripotent Stem Cells
Source: Stem Cell Reports. 2017 Jun 9;8(6):1701–13. doi: 10.1016/j.stemcr.2017.05.013 (PMC5470234; doi:10.1016/j.stemcr.2017.05.013)
Supplement: Document S1. Figures S1–S5 and Tables S1 and S2 [file mmc1.pdf]

Stem Cell Reports, Volume *8*

Supplemental Information

Fetal Therapy Model of Myelomeningocele with Three-Dimensional Skin Using Amniotic Fluid Cell-Derived Induced Pluripotent Stem Cells

Kazuhiro Kajiwara, Tomohiro Tanemoto, Seiji Wada, Jurii Karibe, Norimasa Ihara, Yu Ikemoto, Tomoyuki Kawasaki, Yoshie Oishi, Osamu Samura, Kohji Okamura, Shuji Takada, Hidenori Akutsu, Haruhiko Sago, Aikou Okamoto, and Akihiro Umezawa

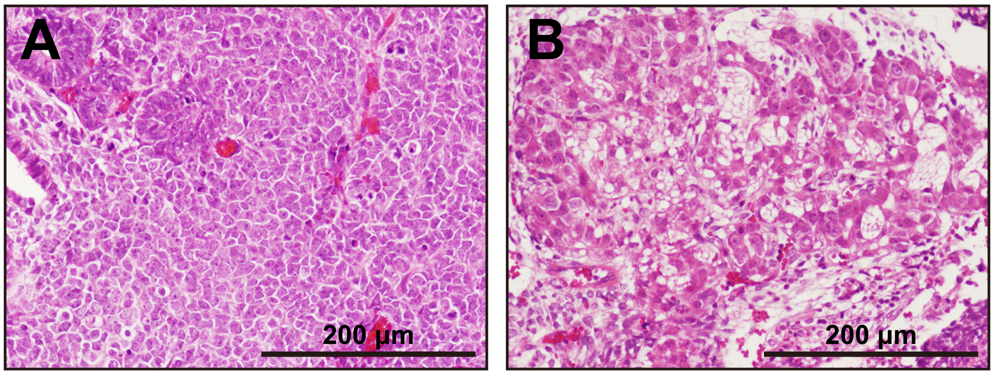

C

Locus	AF-T21-iPSCs			T21-AFC			AF-TTTS-iPSCs		TTTS-AFC	
D3S1358	15	16		15	16		15	16	15	16
TH01	6	7		6	7		6	7	6	7
D21S11	30	31	32	30	31	32	30		30	
D18S51	13	15		13	15		13	15	13	15
Penta_E	12	19		12	19		18		18	
D5S818	12			12			10	12	10	12
D13S317	11	12		11	12		8	12	8	12
D7S820	10	12		10	12		11		11	
D16S539	9	11		9	11		9	11	9	11
CSF1PO	10			10			10	13	10	13
Penta_D	9	10	11	9	10	11	9	13	9	13
AMEL	X			X			X		X	
vWA	14	19		14	19		14	17	14	17
D8S1179	12	13		12	13		10	12	10	12
TPOX	10	12		10	12		8	9	8	9
FGA	22			22			OL	21	OL	21

Supplemental Figure S1. Teratoma formation of in amniotic fluid cells from patients with Down syndrome (AF-T21-iPSC) in vivo. Related to Figure 2.
(A) Neuroblastoma-like tissue. **(B)** Liver tissue with vacuolar structure. **(C)** STR analysis of AF-T21-iPSC, T21-AFC, AF-TTTS-iPSC, and TTTS-AFC. Short tandem repeat (STR) profiling was performed by BEX CO., LTD, Tokyo, Japan. The 16 loci analyzed by the PowerPlex 1.2 system (Promega, Madison, WI, USA) was composed of D3S1358, TH01, D21S11, D18S51, Penta E, D5S818, D13S317, D7S820, D16S539, CSF1PO, Penta D, AMEL, vWA, D8S1179, TPOX, and FGA. T21-AFC and TTTS-AFC were parental cells of AF-T21-iPSC and AF-TTTS-iPSC, respectively.

Supplemental Figure S1

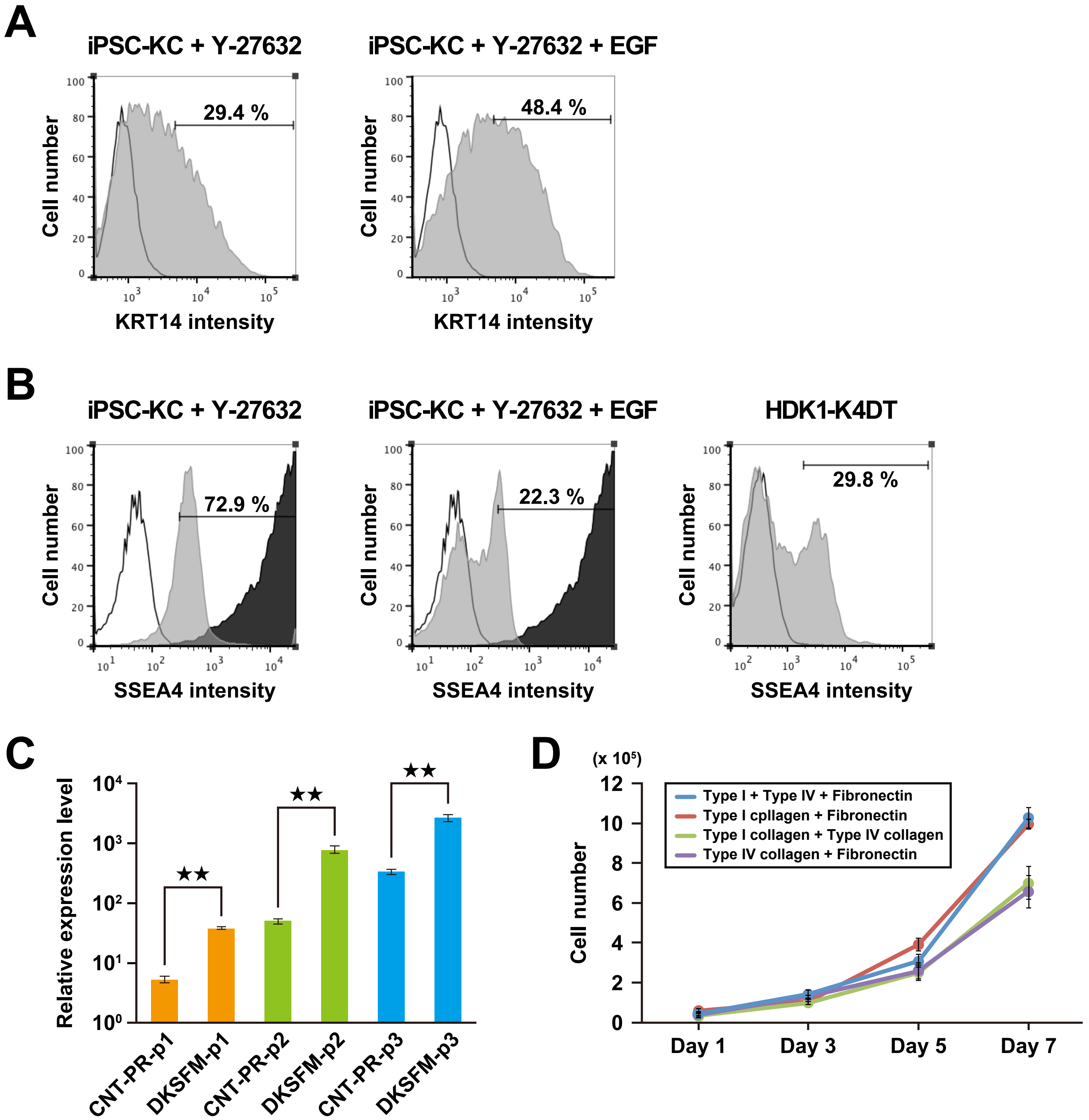

Supplemental Figure S2. Establishment of differentiation protocol of iPSCs into the lineage of keratinocytes. Related to Figure 3.

(A) Flow cytometric analysis of KRT14 in iPSC-KCs at passage 2 (gray) and at passage 0 (open). iPSC-KCs were exposed to Y-27632 and EGF. **(B)** Flow cytometric analysis of SSEA4 in T21-iPSC-KCs. T21-iPSC-KCs at passage 1 were exposed to Y27632 alone or Y27632 and EGF for 1 week and then applied to the flow cytometric analysis. Undifferentiated AF-T21-iPSCs were also shown in black for reference. The SSEA4 reactivity of HDK1-K4DT is displayed for reference. Isotype control is included at each panel. **(C)** qRT-PCR analysis of the *KRT14* transcripts in iPSC-KCs maintained either in a medium of DKSFM or CNT-PR with EGF and Y27632. The *KRT14* gene expression level was higher with the use of DKSFM than with the use of CNT-PR medium. Data are presented as a mean ± SD of three independent experiments. **(D)** Comparison of the cell proliferation of iPSC-KCs on the matrix combinations with type I collagen, type IV collagen, and fibronectin. The combination of “type I collagen and fibronectin” was suitable for the cell proliferation of iPSC-KCs. Values are shown as mean ± standard deviation from three independent experiments.

Supplemental Figure S2

A

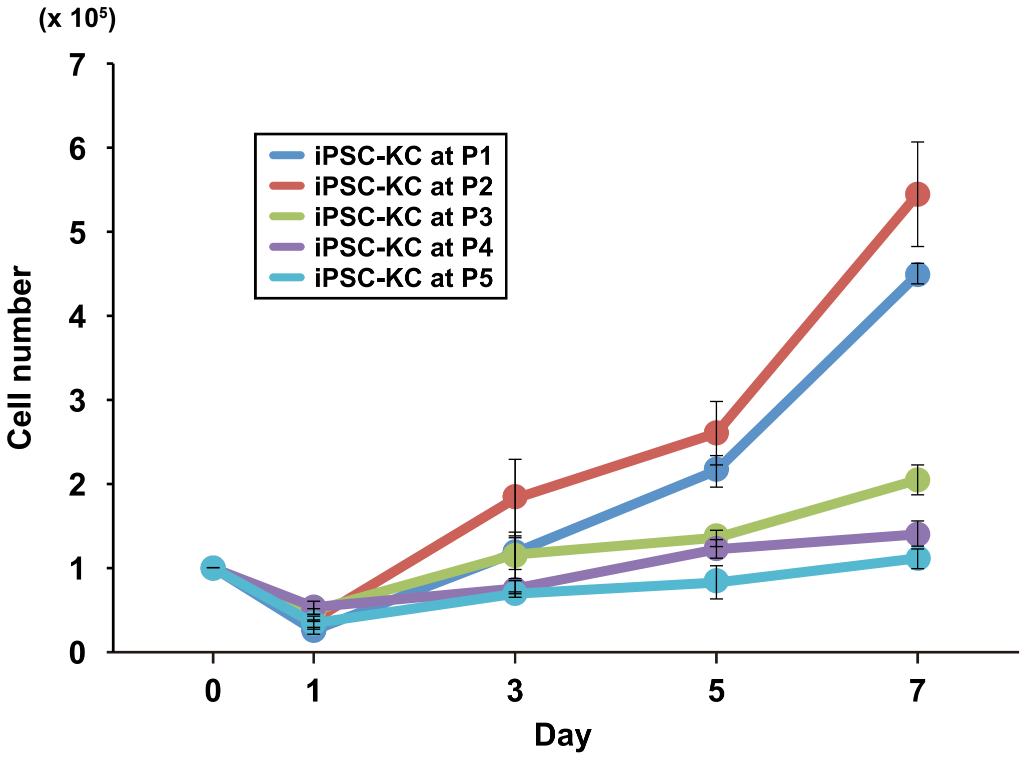

B

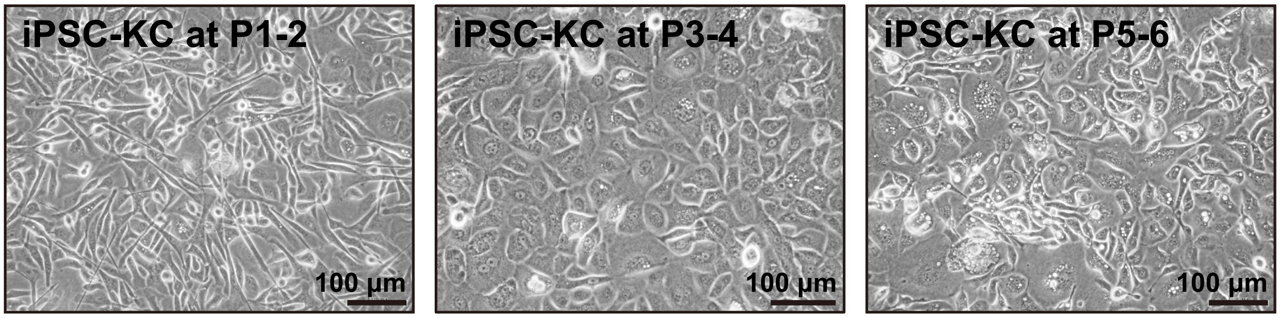

Supplemental Figure S3: Characterization of T21-iPSC-KCs at different passage number. Related to Figure 4. **(A)** The growth rate of T21-iPSC-KCs with the different passage number. Growth rate was reduced as each passage progressed. The initial cell number was 1 × 10^5/well, and the cell number was counted at the indicated days after cell seeding. Values are shown as mean ± standard deviation from three independent experiments. **(B)** Cell morphology of T21-iPSC-KCs in different passage numbers. iPSC-KCs at early passage exhibited spindle cell morphology (iPSC-KC at P1-2) and became keratinocytic (iPSC-KC at P3-4). In later passages, keratinocyte-like cells with a vacuolar degeneration (iPSC-KC at P5-6) were observed. Image of “iPSC-KC at passage 3-4” is identical to “T21-iPSC-KC” in Figure 4A.

Supplemental Figure S3

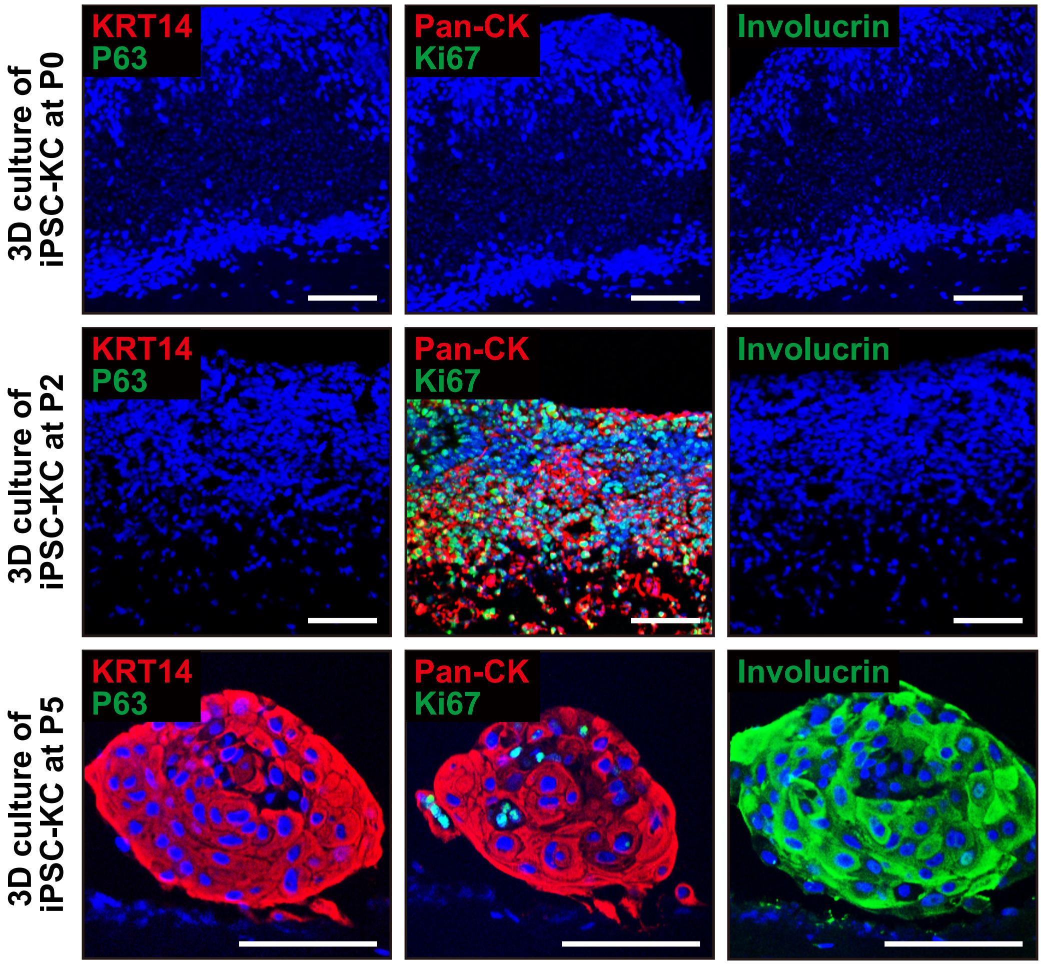

Supplemental Figure S4. Immunohistochemical analysis on three-dimensional (3D) culture of iPSC-KCs at different passages. Related to Figure 4.
iPSC-KCs at passage 0 were negative for KRT14, p63, pan-cytokeratin (Pan-CK), and Ki67. iPSC-KCs at passage 1 became positive for Pan-CK and Ki67 but not for KRT14 and p63. iPSC-KCs at passage 5 were positive for KRT14, Pan-CK, and involucrin but not for p63, indicating terminal differentiation of epidermis in the 3D-culture.

Supplemental Figure S4

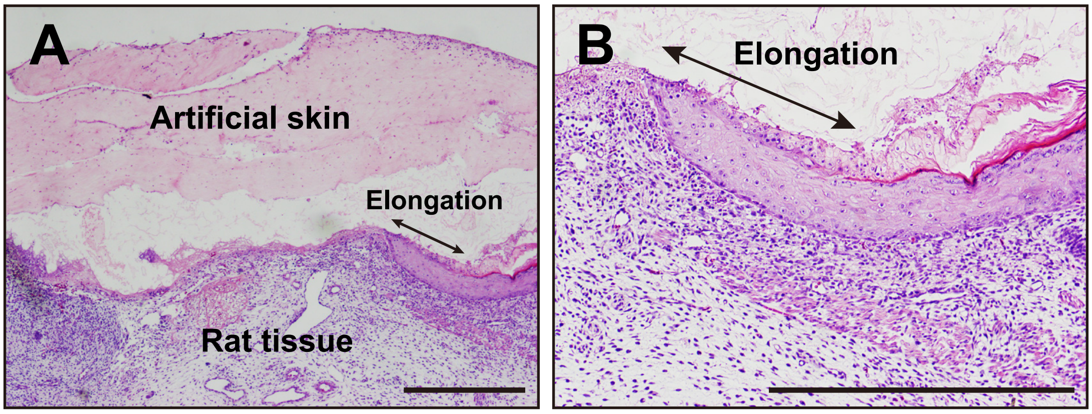

Supplemental Figure S5. Short-term in vivo effect on the regeneration of rat skin defect with iPSC-KC artificial skin. Related to Figure 5.
Epidermal ingrowth from the edge of the MMC defect was observed underneath the artificial skin under low magnification **(A)** and high magnification **(B)**. Scale bar is 500 μm.

Supplemental Figure S5

Supplemental Table S1. List of primers used in this study

Gene	Primer sequence		Aim
OCT3/4	Forward	TGTACTCCTCGGTCCCTTTC	qRT-PCR
	Reverse	TCCAGGTTTTCTTTCCCTAGC	
NANOG	Forward	CAGTCTGGACACTGGCTGAA	qRT-PCR
	Reverse	CTCGCTGATTAGGCTCCAAC	
SOX2	Forward	ATGGGTTCGGTGGTCAAGT	qRT-PCR
	Reverse	GGAGGAAGAGGTAACCACAG	
TERT	Forward	GGAGCAAGTTGCAAAGCATTG	qRT-PCR
	Reverse	TCCCACGACGTAGTCCATGTT	
DNMT3B	Forward	GGAAATTAGAATCAAGGAAATACGA	qRT-PCR
	Reverse	AATTTGTCTTGAGGCGCTTGCC	
KRT14	Forward	GACCATTGAGGACCTGAGGA	qRT-PCR
	Reverse	CATACTTGGTGCGGAAGTCA	
KRT18	Forward	GAGTATGAGGCCCTGCTGAACATCA	qRT-PCR
	Reverse	GCGGGTGGTGGTCTTTTGGAT	
ΔNp63	Forward	GGAAAACAATGCCCAGACTC	qRT-PCR
	Reverse	GTGGAATACGTCCAGGTGGC	
INVOLUCRIN	Forward	GGAGAAAACACAAAGGGATCAG	qRT-PCR
	Reverse	TCCAACAGTTGCTCTTTCTTCA	
FILAGGRIN	Forward	TTCGGCAAATCCTGAAGAATC	qRT-PCR
	Reverse	CTTGAGCCAACTTGAATACCATC	
GAPDH	Forward	TGTTGCCATCAATGACCCCTT	qRT-PCR
	Reverse	CTCCACGACGTACTCAGCG	

Supplemental Table S2. List of antibodies used in this study			
	Class	Company	Dilution
Primary antibodies			
Mouse anti-human OCT3/4	Mouse IgG2b	Santa cruz biotechnology	1/300
Anti-human NANOG	rabbit polyclonal	ReproCELL	1/200
Rabbit anti-human SOX2	rabbit polyclonal	Millipore	1/300
Mouse anti-SSEA-4	Mouse IgG3	Millipore	1/300
Anti-TRA-1-60	Mouse IgM	Millipore	1/300
Anti-TRA-1-81	Mouse IgM	Millipore	1/300
αSMA	Mouse IgG2a	Sigma-Aldrich	1/400
AFP	Mouse IgG1	R&D systems	1/100
Neuron-specific class III beta-tubulin (TUJ-1)	Mouse IgG1	Promega	1/300
Keratin 14 polyclonal antibody	rabbit polyclonal	BioLegend	1/1000
Anti-P63 (4A4) antibody	Mouse IgG2a	Abcam	1/50
Anti laminin 5 antibody	rabbit polyclonal	Abcam	1/200
Keratin 10 polyclonal antibody	rabbit polyclonal	BioLegend	1/1000
Monoclonal Anti-Involucrin antibody produced in mouse	Mouse IgG	Sigma-Aldrich	1/200
Keratin 15 polyclonal antibody	rabbit polyclonal	BioLegend	1/1000
Anti-Ki67 antibody	rabbit polyclonal	abcam	1/100
Anti-Pan-cytokeratin antibody	Mouse IgG1	eBioscience	1/200
Loricrin polyclonal antibody	rabbit polyclonal	BioLegend	1/1000
Anti-filaggrin antibody	Mouse IgG1	abcam	1/50
Stem121, Mouse Monoclonal Antibody Specific for Human Cytoplasmic Marker	Mouse IgG1	TaKaRa	1/1000
PE Mouse Anti-Human CD14	None	BD Bioscience	20 μl/test

FITC Mouse Anti-Human CD19	None	BD Bioscience	20 µl/test
CD29-FITC	None	Beckman Coulter	20 µl/test
CD31-FITC	None	Beckman Coulter	20 µl/test
CD34-PE	None	Beckman Coulter	20 µl/test
CD44-FITC	None	Beckman Coulter	20 µl/test
PE Mouse Anti-Human CD73	None	BD Bioscience	20 µl/test
CD105-PE	None	Beckman Coulter	20 µl/test
FITC Mouse Anti-Human HLA-DR	None	BD Bioscience	20 µl/test
FITC Mouse Anti-Human HLA-ABC	None	BD Bioscience	20 µl/test
FITC Mouse IgG2a, κ Isotype Control	None	BD Bioscience	20 µl/test
PE Mouse IgG1, κ Isotype Control	None	BD Bioscience	20 µl/test
Mouse IgG1 (isotype control)-FITC	None	Beckman Coulter	20 µl/test
Secondary antibodies			
Goat anti-Mouse IgG1 Secondary Antibody, Alexa Fluor® 488	None	Invitrogen	1/1000
Goat anti-Mouse IgG1 Secondary Antibody, Alexa Fluor® 546	None	Invitrogen	1/1000
Goat anti-Mouse IgG2a Secondary Antibody, Alexa Fluor® 488	None	Invitrogen	1/1000
Goat anti-Mouse IgG2a Secondary Antibody, Alexa Fluor® 546	None	Invitrogen	1/1000
Goat anti-Mouse IgG2b Secondary Antibody, Alexa Fluor 568	None	Invitrogen	1/1000
Goat anti-Mouse IgG3 Secondary Antibody, Alexa Fluor 488	None	Invitrogen	1/1000
Goat anti-Mouse IgM Heavy Chain Secondary Antibody, Alexa Fluor 488	None	Invitrogen	1/1000
Goat anti-Mouse IgG (H+L) Secondary Antibody, Alexa Fluor® 546	None	Invitrogen	1/1000
Goat anti-Chicken IgG (H+L) Secondary Antibody, Alexa Fluor® 488	None	Invitrogen	1/1000
Rabbit IgG (H+L) Polyclonal Secondary Antibody for IF, Flow	None	Invitrogen	1/1000
Rabbit IgG (H+L) Polyclonal Secondary Antibody for IF, ICC, Flow	None	Invitrogen	1/1000
